# Temporal reconstruction of a Salmonella Enteritidis ST11 outbreak in New Zealand

**DOI:** 10.1099/mgen.0.001525

**Published:** 2025-10-30

**Authors:** Hugo Strydom, Jackie Wright, Collette Bromhead, David Welch, Ernest Williams, Kerry Mulqueen, Joep de Ligt, Patrick J. Biggs, Shevaun Paine, Sarah Jefferies, Nigel French

**Affiliations:** 1 Enteric Reference Laboratory, Institute of Environmental Science and Research, Upper Hutt, New Zealand; 2 Tāwharau Ora | School of Veterinary Science, Massey University, Palmerston North, New Zealand; 3 Health and Environment Group, Institute of Environmental Science and Research, Christchurch Science Centre, Christchurch, New Zealand; 4 School of Health Sciences, Massey University, Wellington, New Zealand; 5 School of Computer Science, University of Auckland, Auckland, New Zealand; 6 Poultry Industry Association of New Zealand (PIANZ), Auckland, New Zealand; 7 Genomics and Bioinformatics, Health and Environment Group, Institute of Environmental Science and Research, Porirua, New Zealand; 8 School of Food Technology and Natural Sciences, Massey University, Palmerston North, New Zealand; 9 New Zealand Food Safety Science and Research Centre, Massey University, Palmerston North, New Zealand; 10 ESR Health Intelligence and Surveillance, Health and Environment Group, Institute of Environmental Science and Research, Porirua, New Zealand

**Keywords:** Bayesian integrated coalescent epoch plots (BICEPS), marginal approximation of the structured coalescent (MASCOT), New Zealand, outbreak, poultry, *Salmonella* Enteritidis

## Abstract

*A corrigendum of this article has been published full details can be found at* *10.1099/mgen.0.001594.*

Outbreaks caused by *Salmonella* Enteritidis are commonly linked to eggs and poultry meat internationally, but this serovar had never been detected in Aotearoa New Zealand (NZ) poultry prior to 2021. Locally designated genomic cluster *Salmonella* Enteritidis_2019_C_01, was implicated in a 2019 outbreak associated with a restaurant in Auckland. Four Enteritidis_2019_C_01 sub-clusters have since been identified, two retrospectively, in the Auckland region. Authorities initiated a formal outbreak investigation after genomically indistinguishable *S*. Enteritidis was isolated from the NZ poultry production environment. This study analysed 231 *S*. Enteritidis genomes obtained from the outbreak using Bayesian phylodynamic tools to gain insight into the outbreak’s dynamics and origin. We used Bayesian integrated coalescent epoch plots to estimate the change of the Enteritidis ST11 population size over time and marginal structured coalescent approximation to estimate transmission between poultry producers. We investigated human and poultry isolates to elucidate the time and location of the most recent common ancestor of the outbreak and transmission pathways. The median most recent common ancestor was estimated to be February 2019. We found evidence of amplification and spread of strain Enteritidis_2019_C_01 within the poultry industry, as well as transmission events throughout the production chain. The intervention by the public health and food safety authorities coincided with a drop in the effective population size of the *S*. Enteritidis ST11 as well as notified human cases. This information is crucial for understanding and preventing the transmission of *S*. Enteritidis in NZ poultry to ensure poultry meat and eggs are safe for consumption.

**Impact Statement**

This study provides insight into the transmission dynamics and structure of the first-ever *Salmonella* Enteritidis outbreak within the New Zealand poultry industry and human population. Disease models in combination with genomic and epidemiological data provide the poultry industry, regulators and public health investigators with new insight into transmission pathways for zoonotic diseases in the industry and should in the future allow for prompt interventions in the event of related human foodborne disease outbreaks. This study further demonstrates the utility of approximate structured coalescent modelling in a structured localized bacterial disease outbreak but also shows that care should be taken when analysing and interpreting the data and model output due to model assumptions that are seldom true in real-life outbreaks. Furthermore, this study provides new, publicly available *S.* Enteritidis genomic data and evaluates whether interventions introduced by food safety and public health authorities were impactful. It guides future consideration to avoid the spread of bacterial pathogens within a primary industry.

## Data Summary

The authors confirm all supporting data, code and protocols have been provided within the article, appropriate repositories or in supplementary data files. All relevant metadata are available in the supplementary material. Genome sequences have been deposited in the National Center for Biotechnology Information Sequence Read Archive public database under BioProject PRJNA720150.

## Introduction

Salmonellosis is a significant cause of gastrointestinal disease in humans in Aotearoa New Zealand (NZ) and worldwide, the majority of which is caused by *Salmonella enterica* subs. *enterica* [1]. Chicken eggs and poultry meat are important sources of salmonellosis internationally [2,3], although epidemiological evidence suggested that eggs have not been an important pathway for human salmonellosis in NZ [4]. *S. enterica* subs. *enterica* serovar Enteritidis (*S*. Enteritidis) is the predominant serovar causing egg and poultry meat outbreaks in Europe and North America [2,5]. *S*. Enteritidis is a poultry-adapted serovar that has a higher potential than other serovars for transovarian transmission, involving transmission from the chicken reproductive tract into egg contents before full egg formation [2,3]. Furthermore, transovarian potential has been considered more likely for certain *S*. Enteritidis phage types, such as phage type 8 (PT8) [6,7]. Between 2015 and 2018, the largest known salmonellosis outbreak in Europe was reported, involving 1,209 cases in 18 countries, caused by *S*. Enteritidi*s* contaminated eggs [8,9]. The European Food Safety Authority reported another outbreak of *S*. Enteritidis sequence type (ST) 11 involving 193 cases in 8 European Union countries, including the UK, between May 2018 and December 2020, where poultry meat products were implicated [5]. Furthermore, Agriculture Victoria reported *S*. Enteritidis in Australian poultry in May 2018 when it was detected in several New South Wales and two Victoria egg layer farms, which until then had been considered free of *S*. Enteritidis [10,11].

Historically, sporadic cases of salmonellosis caused by *S*. Enteritidis ST11 occurring in NZ were associated with overseas travel rather than domestically acquired infection. The first human clinical case of salmonellosis caused by locally designated strain *S*. Enteritidis_2019_C_01 (henceforth SE-19C01) was reported in May 2019. A cluster of SE-19C01 involving 17 confirmed and 21 additional probable cases occurred in an Auckland restaurant in October 2019 [12]. The source was later considered likely to have been a raw egg dessert. Strain SE-19C01, identified as ST11 and PT8, has since been implicated in four major sub-clusters, the majority occurring in the Auckland region [12,13]. In February 2021, the same strain was isolated by the poultry industry from a raw broiler chicken carcass sampled at the end of primary processing by a large-scale poultry meat processor during routine National Microbiology Database testing [6,14]. Broiler is an industry term used for young chickens raised for the sole purpose of meat consumption. Until this time, there had been no detection of *S*. Enteritidis reported in NZ poultry [12]. The resulting poultry investigation led by the NZ Ministry for Primary Industries (MPI) resulted in the isolation of SE-19C01 in environmental samples from various sources, including broiler and layer poultry flock production environments, as well as a hatchery that supplied the infected layer and broiler farms. SE-19C01 was also detected from subsequent environmental samples (in 2020 and 2021) from the layer farm that supplied eggs to the Auckland restaurant involved in the 2019 outbreak. A formal response jointly led by MPI and the NZ Ministry of Health (MoH) was initiated in April 2021 [6]. The working assumption was that a hatchery, a major supplier of day-old chicks for the broiler and egg industries in NZ, was the source of infection in downstream operations [12]. Initial detections in the traceback investigation were from a broiler producer and a rearer company supplied by the hatchery on the same day [12].

An emergency control scheme to temporarily regulate the poultry production supply chain and manage risk to public health was implemented in October 2021 [12]. Long-term regulation was introduced in October 2022 that required poultry producers to operate under a Risk Management Programme no later than 1 November 2023 [15]. More importantly, it required testing of all poultry production environments for *S*. Enteritidis. In conjunction with the formal response, MPI and National and Regional Public Health Service teams generated a series of media releases emphasizing good food safety practices when consuming eggs [16,17].

Phylodynamic modelling provides unique insights into the epidemiological dynamics of infectious diseases [18]. Measurably evolving genomes from pathogens can be used to reconstruct a dated phylogeny including parameters of epidemiological interest [19]. Phylodynamic methods are effective in inferring transmission events [20], population size dynamics [21] and pathogen population structure [22]. These methods have previously been used to study the transmission of *Salmonella* [9,23, 24]. Many phylodynamic methods are based on coalescent models, which relate the shape of the phylogenetic tree to the size of the population from which samples are drawn, allowing population size dynamics to be inferred using sampled genomes [25].

We use core SNP analysis in combination with two recent phylodynamic methods, BICEPS [26] and MASCOT [27], to better understand the evolutionary trajectory of a *S*. Enteritidis strain SE-19C01 that was at the source of a major outbreak in NZ. The BICEPS model allows for inference of the change in effective population size over time, to better understand the scope of the outbreak as well as the impact of interventions introduced by regulatory agencies. The MASCOT model considers a structured population where each individual is in one of a finite number of demes with migration between demes. It allows concurrent estimation of the phylogeny along with deme population sizes and ancestral locations of lineages. Our analyses included both human and poultry environmental isolates to estimate the time of the most recent common ancestor (MRCA) and transmission pathways within the poultry production environment. This type of information and analysis is crucial for ongoing monitoring of emerging pathogens in poultry, for deciding whether current transmission of *S*. Enteritidis in the poultry production chain is continuing, and to enable timely responses in the event of other human food-borne disease outbreaks.

## Methods

### Dataset

Salmonellosis is a notifiable disease in NZ [28]. Laboratories are required to culture all human clinical samples where culture-independent diagnostic testing was used to detect *Salmonella*. All isolates should be referred to the Enteric Reference Laboratory (ERL) at the Institute of Environmental Science and Research (ESR) for phenotypic and genotypic characterization [29]. The White-Kauffmann-Le Minor scheme was used to subtype *Salmonella* isolates received by ERL by serovar [30]. The serovar designation was affirmed for all *Salmonella* isolates submitted for sequencing using SISTR [31]. Human isolates included in this study were those submitted to ERL between May 2019 and May 2022, which were identified as *S*. Enteritidis ST11 by multilocus sequence typing (MLST) [32,33] and assigned the cluster ID SE-19C01 using SnapperDB [34] against the *S*. Enteritidis reference genome AM933172 [35]. Additionally, all isolates from poultry sources submitted to ERL as part of the outbreak response (June 2020 to November 2021) were included in this study. Lastly, poultry isolates of *S*. Enteritidis submitted to ERL according to the MPI Emergency Control Scheme [36] were stratified based on collection site and collection date, and 38 samples were randomly selected. Poultry producers were anonymized by allocating a letter (A to H) for each producer. Producer A is a hatchery; producers B, C, E and H are egg producers (chickens referred to as layers); and producers D, F and G are broiler producers (broilers). The total dataset included 126 human isolates, 98 poultry isolates (97 with a known source) and seven other isolates arising from samples linked to *S*. Enteritidis-positive poultry operations (two hedgehogs, two cats, a cow, a dog and a goat) (Table S2). The hedgehogs were trapped in the vicinity of poultry hatchery A, and environmental samples were collected as part of monitoring.

### Whole-genome sequencing

The ESR Sequencing Facility performed DNA extraction, library preparation and whole-genome sequencing. DNA extractions were performed using a Chemagic 360 platform (Perkin Elmer) and DNA library preparation using the Nextera XT DNA Preparation (Illumina) Kit. The sequencing was performed on the NextSeq 550 Illumina platform, with the use of the NextSeq 500/550 Mid Output Kit v2.5, producing on average 4.3 million reads (2×150 paired end) per isolate.

### Genome assembly and annotation

Genome sequence trimming, *de novo* assembly and quality assessments were performed using the Nullarbor v2.0.20191013 pipeline [37]. The Nullarbor pipeline implements Trimmomatic v0.39 [38] to remove adaptors, low-quality bases and reads, as well as SPAdes v3.13.1 [39,40] for assembly. Taxonomic classifications were confirmed using Centrifuge v1.0.4 [41] and Kraken v1.1.1 [42]. Assembled genomes were annotated using Prokka v1.14.5 [43].

### Genotypic bacterial characterization

Bacterial characterizations were performed on draft genome assemblies using the BakCharak v3.0.4 [44] pipeline. This pipeline implements ABRicate v1.0.1 [45] to detect antimicrobial resistance genes using National Center for Biotechnology Information AMRFinderPlus v 3.11.11 [46] and detect plasmid compatibility groups using PlasmidFinder [46]. This pipeline implements sistr cmd v1.1.1 [31], mlst v2.23.0 [33] and PubMLST schemes [47] for 7-gene MLST typing and Platon v1.6 [48] for plasmid prediction.

### Multidimensional scaling

A total of 1,476 *S*. Enteritidis ST11 assembled genomes were downloaded from PubMLST [47]. Only isolates with metadata that included the country of origin were included (*n*=59 countries). The core genome multilocus sequence typing (cgMLST) profiles of these isolates and NZ isolates were determined using CHEWBBACA [49], and a distance matrix representing the pairwise dissimilarity in allele profiles was generated (in which zero represented identical allele profiles and one represented no shared alleles). These were analysed using metric multidimensional scaling (mMDS) using the mds command in the R package smacof with no dissimilarity transformation (i.e. type=‘ratio’) [50].

### SNP identification

The Nullarbor pipeline implements the Burrows–Wheeler Alignment tool (BWA MEM v0.7.17-r1188) [51] for alignment, SAMtools v1.9 [52] to compare sequence data against the *S*. Enteritidis reference genome AM933172 [35] and Snippy v4.4.3 [53] to identify core SNPs. Following alignment, recombinant regions were detected and removed using Gubbins v.3.2.1 [54], the reference sequence was removed and SNPs were extracted using SNP-sites v.2.4.1 [55]. A pairwise SNP distance matrix was generated for the resulting sequence alignment using snp-dists v.0.6.3 [56]. A maximum likelihood (ML) phylogeny was inferred for 163 core SNPs, using IQ-TREE [57] and ModelFinder [58] to identify the best nucleotide substitution model. A K3*P*+ASC substitution model was used with 1,000 bootstraps. The midpoint rooted ML tree was visualized using Interactive Tree of Life (iTOL) v6.5.8 [59]. Hierarchical clustering was performed using RhierBAPs v1.0.1 [60].

### Phylogenetic reconstruction

To test for a temporal signal, a linear regression of root-to-tip genetic distance (ML) against sampling times for each tip was generated using TempEST v1.5.3 [61] and BactDating [62] in triplicate, with a different seed used in each iteration. In order to formally test for a temporal signal, two runs of Bayesian ancestral dating models were compared, with and without the sampling dates set equal, using the ‘bactdate’ and ‘modelcompare’ functions in BactDating [62]. Both models were additive relaxed clock models fitted using the ‘bactdate’ command. Model 1 was fitted to the dataset with tip dates equal to the sampling dates. Model 2 was fitted to the dataset with all sampling dates forced to be equal (date=2020). The two models were then compared using the deviance information criterion (DIC) method [63]. Recombinant regions were detected and removed using Gubbins v.3.2.1 [54]. The reference sequence was manually deleted, and SNPs were extracted using SNP-sites v.2.4.1. All isolates were treated as one bacterial population.

A dated phylogenetic tree and evolutionary rates were inferred based on 163 SNPs and the count of invariant sites using BEAST2 v2.7.4 [64]. Analysis of runs with HKY, GTR and bModelTest substitution models showed no significant differences between estimated parameters of interest (data not shown). HKY [65] was selected as it is the simplest and least computationally demanding model. To determine the most suitable clock and population model combination, eight BEAST runs consisting of combinations of two clock models – namely a strict clock and an optimized relaxed clock – and four population models, namely constant population, exponential growth, Bayesian skyline and BICEPS runs, were prepared. Nested sampling v1.2.1 [66] was used to determine the best clock and population model combination. The strict clock in combination with either the Bayesian skyline model or the BICEPS model was indistinguishable from each other and had a marginal likelihood greater than other model combinations tested. The BICEPS model was chosen as it is computationally less demanding. The clock rate prior was based on a previously published estimate [67] using a Log Normal distribution, with a mean of 2.5×10^−7^ and a sd of 0.5. The prior for effective population size was a gamma (0.5,1) distribution. The BICEPS model was fitted using a Markov chain Monte Carlo (MCMC) run for 40 million iterations sampled every 4,000 iterations.

Transmissions between poultry producers were inferred using the marginal structured coalescent approximation model MASCOT v.3.0.7 [27] using the same clock rate prior, a Log Normal population size prior (NeConstant), and an exponential prior (mean=1) on migration rates. Only poultry isolates for which the source was known were included (*n*=89). In addition, ten randomly selected human clinical isolates were included. Isolates from separate poultry producers were treated as sub-populations (demes) in a structured bacterial population. All isolates from humans were considered as part of a single sub-population within the same structured population. An MCMC sampler was run for 30 million iterations and sampled every 3,000 iterations. Convergence and model parameters were visualized using Tracer v1.7.2 [68]. Both models were run in triplicate with different random seeds, and we ensured all effective sample sizes were above 200. In addition, we performed a permutation test with three random independent chains permutated between subpopulations.

The BICEPS and MASCOT trees were summarized using TreeAnnotator v2.7.4 [69], based on median values. For the BICEPS model, a maximum clade credibility (MCC) tree was generated. However, due to parameter complexity, a conditional clade distribution (CCD) tree [70] was generated for the MASCOT model. The summary trees were visualized using FigTree v1.4.4 [71].

## Results

### Analysis of genomes

Whole-genome sequencing analysis was performed on 231 isolates. An average of 4.3 million reads was obtained with an average G+C content of 51.9 mol% and a read depth above 47 (average=132). Assembled genomes produced an average of 30 contigs (all below 103), a genome size of 4.7 Mb and an N50 above 60,000 bp. These fulfil suggested quality control requirements for further analysis [72]. MLST analysis confirmed all sequenced isolates to be *S*. Enteritidis ST11.

### Genotypic characterization

The antimicrobial genes coding for the *Salmonella* specific resistance–nodulation–division efflux pump MsdAB were detected. It has been reported that MdsABC conferred resistance to novobiocin, nitrofurans and several toxic chemicals [73,78]. Genes coding for the transporter periplasmic adaptor subunit MdsA and the transporter permease subunit MdsB were detected in all samples. However, genes coding for MdsC or TolC, the outer membrane component required for the MdsAB efflux pump to function [74], were not detected. The gold/copper-translocating P-type ATPase GolT and the Au(I) sensory transcriptional regulator GolS have been detected for all isolates tested, suggesting a decreased susceptibility to gold and copper. For one isolate, an additional gene coding for tellurium resistance-associated protein TerZ was detected. The replicons for plasmid incompatibility groups IncFIB(S)_1 and IncFII(S)_1 were detected for 226 isolates tested. For three of these isolates, the additional replicon for colicin family plasmids ColpVC_1 was detected, as well as IncFII(29)_1 and pUTI89 for one other and pSL483_1 for another. For one isolate, additional replicons for Col(BS512)_1, Col(MG828)_1, Col156_1, ColpVC_1 and ColRNAI_1 were detected. With the data available, we cannot conclude if replicons identified on separate contigs belong to the same plasmid [46]. For five isolates, no plasmids were predicted.

### Multidimensional scaling

NZ isolates of SE-19C01 were compared with Enteritidis ST11 international genomes using cgMLST and mMDS and showed that SE-19C01 clustered independently from all other genomes in the dataset (Fig. S1, available in the online Supplementary Material). The most similar other NZ genome was a traveller from Turkey. This isolate was most similar to other isolates from Turkey and the UK.

### Maximum likelihood

**Fig. 1.**
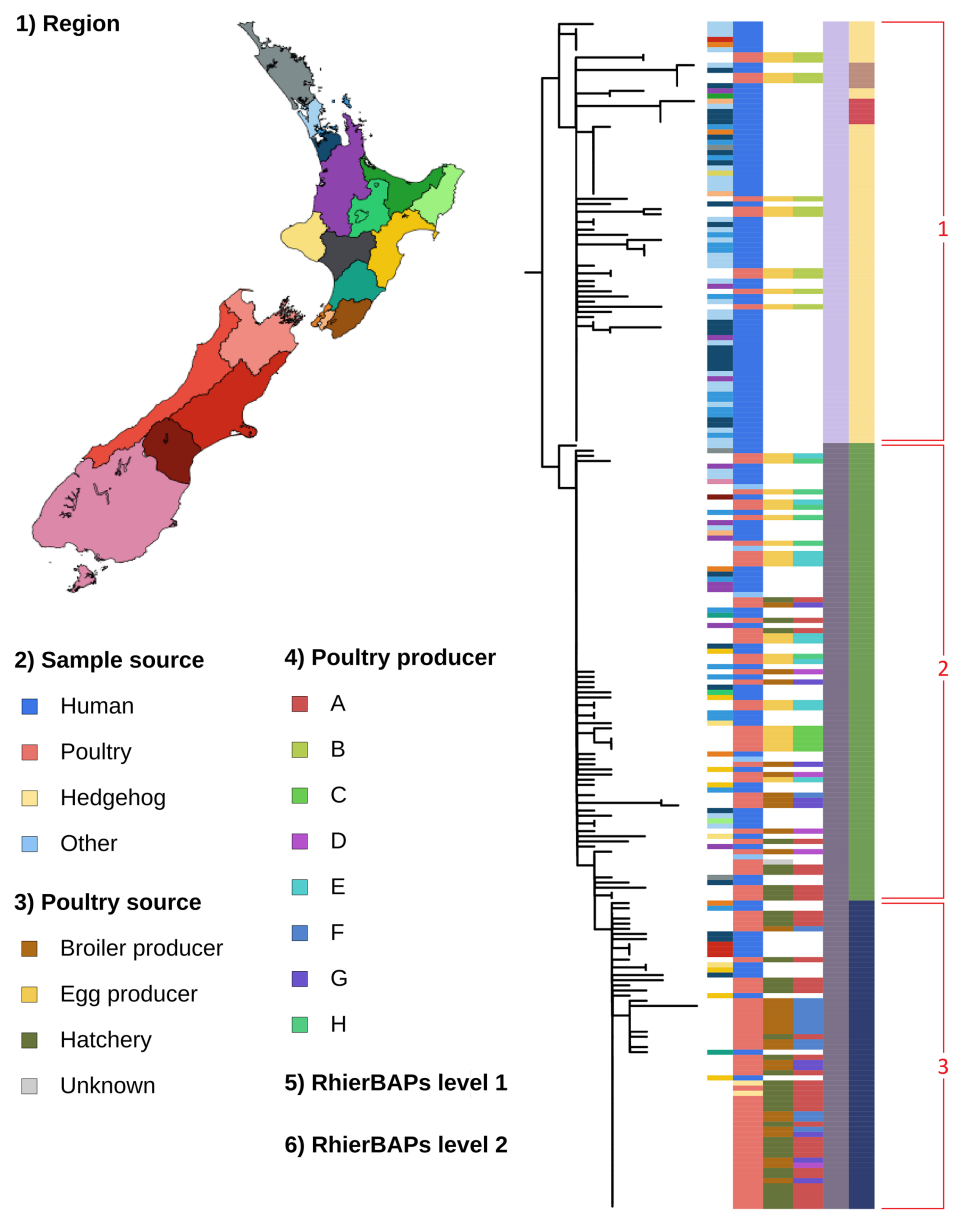
The midpoint rooted ML tree constructed using 163 core SNPs amongst isolates of *S.* Enteriditis ST11 SE-19C01 collected during an outbreak investigation and the subsequent emergency order and control scheme implemented by the NZ Ministry for Primary Industries. Colour bars represent (1) the public health region where human cases were notified, (2) the sample source, (3) the poultry source, (4) the anonymized poultry producer, (5) clades inferred using RhierBAPs – level 1 and (6) RhierBAPs – level 2. Red numbered partitions show clades inferred by RhierBAPs.

The maximum pairwise core SNP distance between all isolates was 18 based on the SNP distance matrix generated. Hierarchical clustering (RhierBAPs) revealed three distinct clades, as well as two sub-clades within clade 1 (Fig. 1). Clade 1 consisted of isolates sampled from human sources and from egg producer B only, but no isolates sampled from hatchery A or any broiler producer (Fig. 1). Human isolates included those associated with the Auckland restaurant cluster as well as various other human clinical clusters around the Auckland region. Clade 2 included human isolates, hatchery A, egg producers E, C and H, and broiler producers D, F and G (Fig. 1). Clade 2 also included isolates sampled from domestic animals associated with infected poultry operations (two cats, a cow, a dog and a goat). Clade 3 comprised isolates sampled from human cases, hatchery A and broiler producers F and G and isolates obtained from two hedgehogs trapped near hatchery A, but no isolates sampled from any egg producer (Fig. 1). There was zero core SNP difference between the isolates from the hedgehog and isolates from hatchery A, and broiler producers F and G.

### Unstructured coalescent

A linear regression of root-to-tip genetic distance against sampling dates for each tip using the R-squared method produced a correlation coefficient of 0.82 with an *R*-squared value of 0.67, indicating a correlation between genetic divergence and sampling time (Fig. S2) suitable for BEAST analysis. The temporal signal was supported by the Bayesian inference model BactDating; the *R*-square value for the root-to-tip regression was 0.7 (*P*<0.0001), and the DIC values for model comparison showed that the model using the sampling dates (model 1) was consistently preferred over the model with the dates set equal (model 2). Model 1 had a DIC of 762.57, and model 2 had a DIC of 842.46. As the DIC was markedly lower for model 1, it was concluded that model 1 was ‘definitely better’ than model 2.

**Fig. 2.**
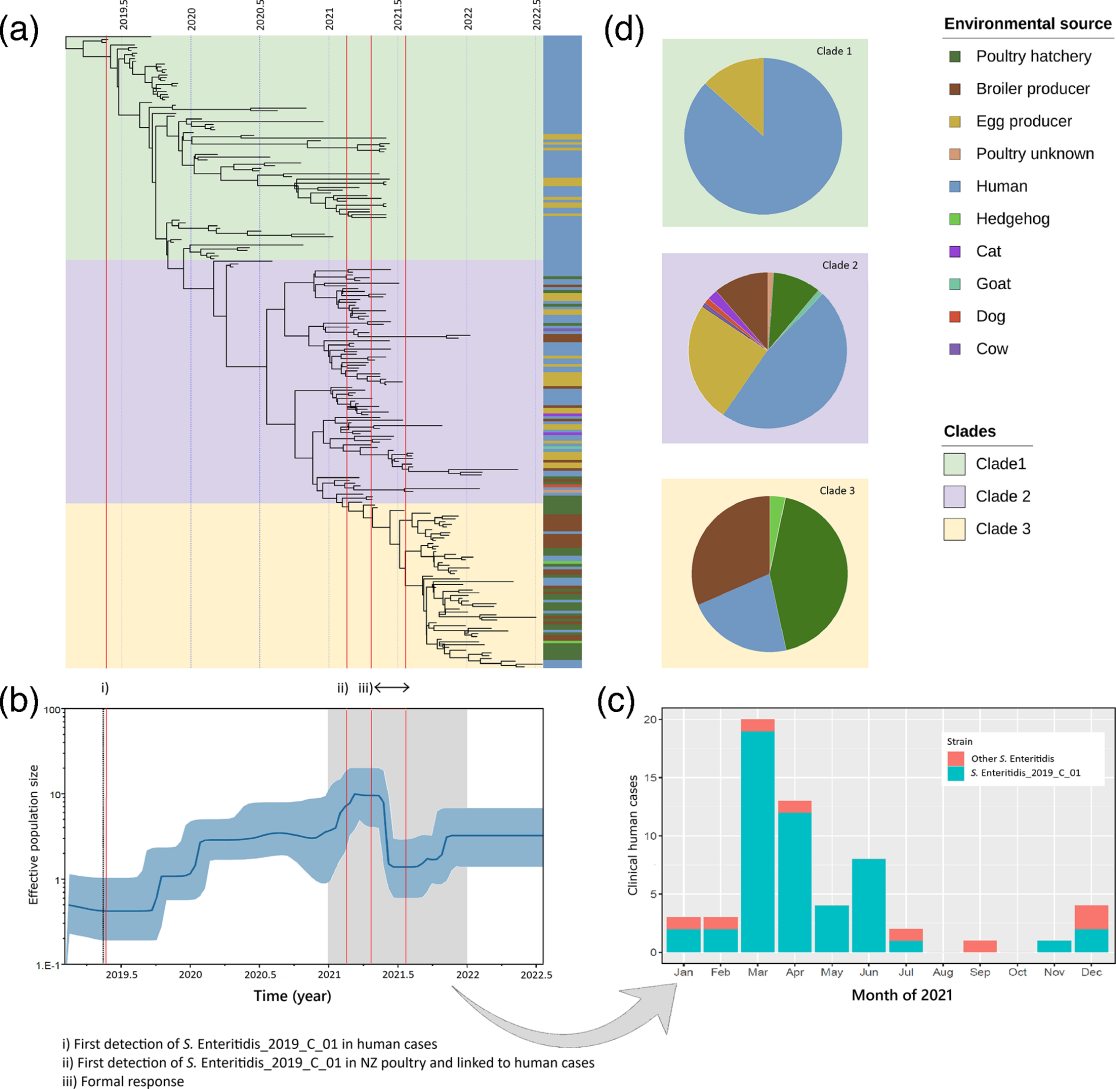
(a) Maximum clade credibility tree generated using BICEPS. The total number of isolates included 126 clinical human isolates, 98 poultry isolates and 7 other isolates associated with the poultry environment (including 2 each from hedgehogs and cats and 1 each from a cow, dog and goat). The tree is overlaid with colours denoting the three clades determined using RhierBAPs. (b) Historical changes in the effective population size of *S.* Enteritidis SE-19C01. The dark blue line is the median of the estimated effective population size. The blue shaded areas are the upper and lower bounds of the 95% HPD interval. The *x*-axis is the time in years in decimal dates and the *y*-axis is on a log scale. (c) Notified human cases of salmonellosis caused by *S.* Enteritidis during 2021. (d) Pie graphs showing the proportion of isolates from each source within clades of the maximum clade credibility tree (**a**) using the overlayed colours for the corresponding RhierBAPs clade.

For Bayesian analysis using BICEPS, three independent chains with different random seeds converged on similar posterior distributions. BICEPS inferred a mean substitution rate of 5.7×10^−7^ substitutions per site per year with a 95% highest posterior density (95% HPD) interval of 4.4×10^−7^ to 7.0×10^−7^. The median root height was estimated at 3.46 years (95% HPD, 3.18–4.06). Since the most recent isolate was sampled on 10 May 2022, that equates to the median MRCA estimated to be February 2019 (95% HPD, June 2018–May 2019) (Fig. 2a). Except for one isolate sampled in 2020, all poultry isolates were sampled from 2021 onwards. BICEPS analysis shows an exponential increase in the effective population size starting mid-2019 at the beginning of the outbreak, followed by a significant decrease in mid-2021 (Fig. 2b). Notified human clinical cases of *S*. Enteritidis increased in March 2021, followed by a steep decline starting in April 2021 (Fig. 2c).

### Approximate structured coalescent

For Bayesian analysis using MASCOT, three independent chains with different random seeds converged on similar posterior distributions. In addition, permutated runs produced clearly distinguishable results (Fig. S3), showing that phylodynamic analysis was not overly biassed by raw case counts from each source. The MASCOT model produced median posterior values of the effective population sizes (NeConstant) for poultry producers A, B, C, D, E, F, G, H and humans were 0.19, 2.58, 0.14, 1.58, 0.62, 0.17, 0.34, 0.53 and 1.50 (Fig. S4), respectively. The mean substitution rate was estimated to be 5.0×10^−7^ substitutions per site per year (95% HPD, 3.6–6.7×10^−7^). The median root height was estimated at 3.53 years (95% HPD, 2.69–4.65). Since the most recent isolate was sampled on 18 March 2022, that equates to the median MRCA estimated to be September 2018 (95% HPD, July 2017–July 2019).

**Fig. 3.**
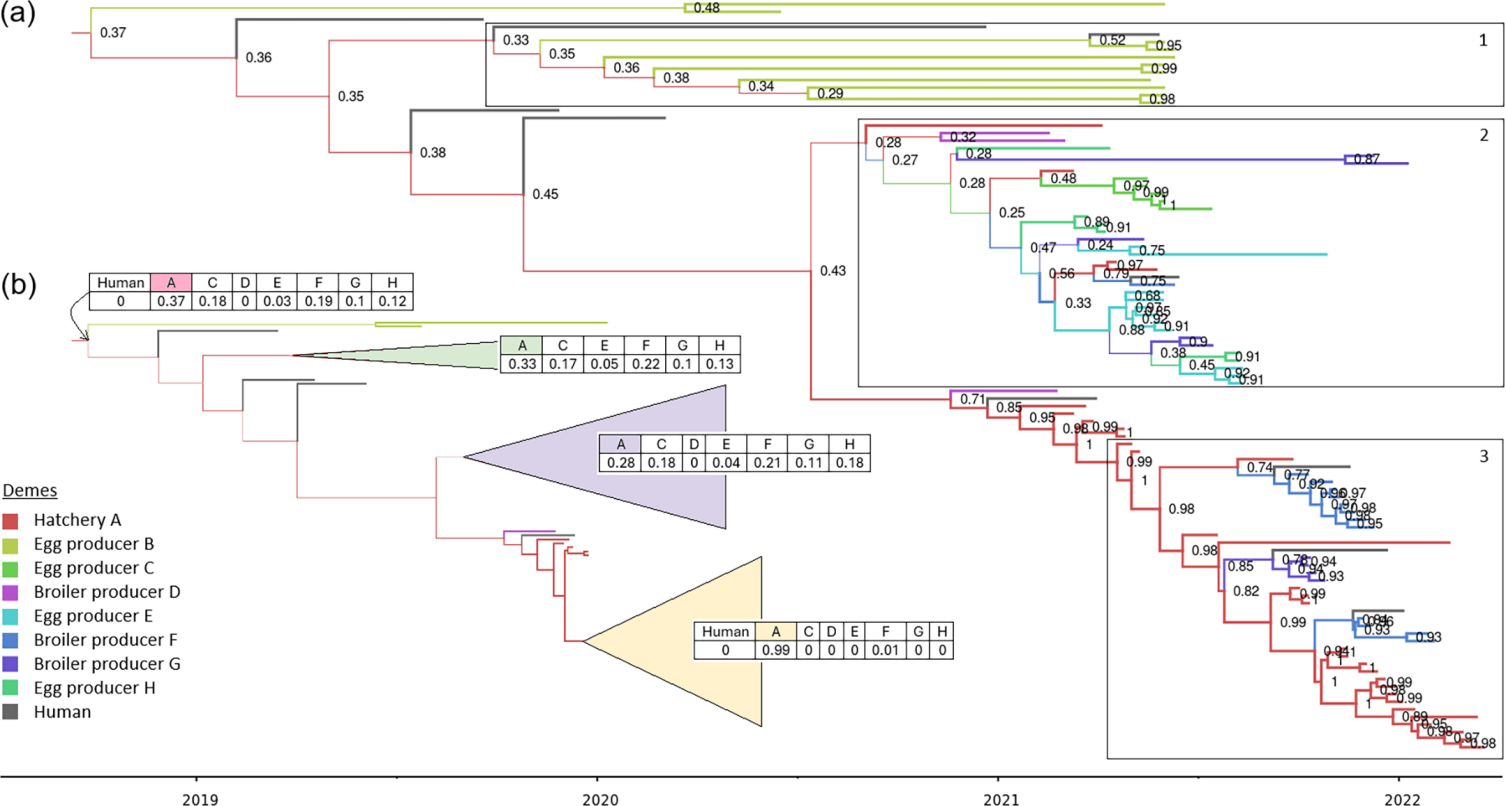
(a) A conditional clade distribution tree using MASCOT, of *S.* Enteritidis SE-19C01 isolates obtained from implicated poultry producers and ten clinical human isolates. Branches are coloured according to their most probable ancestral state of sister branches. Nodes are labelled with the maximum posterior probability of the ancestral state. (b) Deme sets and the corresponding posterior probability sets for the root and three clades of interest.

The maximum posterior probability for a transmission event denotes the location of an ancestral lineage for a clade with the highest probability out of all locations (poultry producers A–H). The model suggests that the most probable ancestral root for the isolates included in this study to be hatchery A with the maximum posterior probability of 0.37, followed by broiler producer F with a posterior probability of 0.19 (Fig. 3b). Transmission from hatchery A to egg producer B was inferred to have occurred on several occasions in early 2019, with maximum probability ranging from 0.29 to 0.37 (Fig. 3a, clade 1). When eight additional isolates from clinical human cases associated with the Auckland restaurant cluster were included in the analysis, hatchery A was inferred as the ancestral lineage with maximum probability (Fig. S5).

The estimates for the ancestral location for clade 2 have low posterior probability, with hatchery A having the maximum posterior probability of 0.28, followed by broiler producer F with a probability of 0.21 (Fig. 3a, clade 2). This clade consists of isolates from hatchery A; egg producers C, E and H; and broiler producers D, F and G (Fig. 3a, clade 2). The ancestral location for clade 3 is estimated to be with hatchery A with high certainty (maximum posterior probability of 0.99) followed by broiler producer F with a posterior probability of 0.01 (Fig. 3a, clade 3). This clade consists of isolates from hatchery A and broiler producers F and G.

**Fig. 4.**
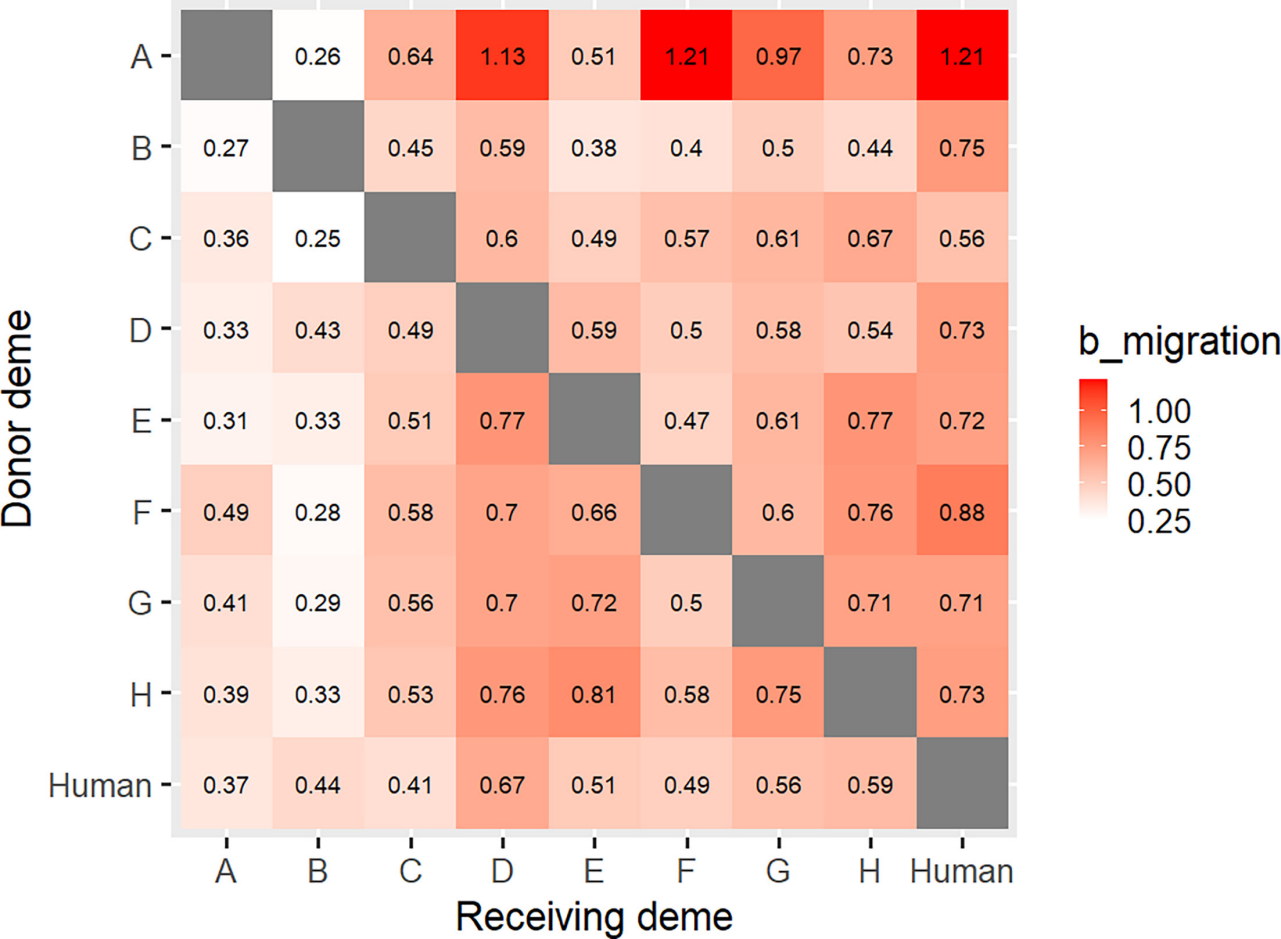
Matrix showing b_migration (*x*-axis) to (*y*-axis) obtained using MASCOT. Deme A is a poultry hatchery, demes B, C, E and H are egg producers and demes D, F and G are broiler producers.

MASCOT estimates backwards-in-time migration rates (denoted b_migration) where the backward rate from *x* to *y* can be interpreted as *y* being the donor deme and *x* being the receiving deme. The median inferred b_migration rates show that A is the dominant donor deme, with only deme B not having deme A as the leading, or close to the leading donor (Fig. 4, Fig. S6).

### Euclidean distances

Some isolates sampled from producers A, F and G were indistinguishable from each other (0 SNP difference). These include hedgehog isolates sampled in the vicinity of producer A that were indistinguishable from isolates sampled at producer A (Fig. 1, clade 3). Producer F and producer G have operations at multiple sites, while all operations for producer A were within a 1 km radius. To evaluate if the spread of SE-19C01 could be facilitated by hedgehogs or rodents such as rats, Euclidean distances were calculated between poultry sites (Table S1). Distances ranged between 1 and 413 km.

## Discussion

### Inferred phylogenies suggest a recent single introduction

To better understand the transmission pathways of a *S*. Enteritidis strain SE-19C01, the cause of a major outbreak in NZ, we used core SNP analysis in combination with BICEPS and MASCOT Bayesian analysis. The isolates included in this study from an outbreak of *S*. Enteritidis ST11 were all within 18 SNPs of each other. On its own, this genetic similarity suggests that all isolates come from a single recent introduction into the poultry environment that has propagated locally in the poultry chain over time. We have shown using a cluster analysis that this SE-19C01 strain was distinct from all international *S*. Enteritidis ST11 genomes tested. The monophyletic nature further supports a single introduction.

Phylodynamic analyses of the data further support this hypothesis and provide additional information about the evolution, population dynamics and transmission of the outbreak strain. We fitted two phylodynamic models to study distinct aspects of the data. The BICEPS and MASCOT models are both tree priors based on the coalescent which relates the dynamics of the outbreak strain to the timings and shape of the phylogenetic tree. BICEPS is a non-parametric model of a population that can change in size through time but is otherwise well-mixed. It is like the Bayesian Skyline [79] and Skyride [80] family of models. MASCOT approximates the structured coalescent where a population is divided into multiple well-mixed demes with migration between demes. Each deme has a constant population size, which can be estimated. The same clock rate prior was used for both these models based on previously published data [67]. Each model answered a different question; the BICEPS model addressed questions relating to ancestral dates and population dynamics, whereas the MASCOT model addressed questions relating to migration between sub-populations.

The phylogenies inferred under the BICEPS and MASCOT models have similar root ages [1]: February 2019 (95% HPD, June 2018–May 2019) for BICEPS and September 2018 (95% HPD, August 2017–July 2019) for MASCOT. Thus, both point to a time of introduction shortly prior to the first human case in May 2019. Given the misspecification in both these models and the greater flexibility in the BICEPS model, we suggest BICEPS is the more reliable estimate. We address model misspecification in more detail later in this discussion.

The inferred mean substitution rate is 5.7×10^−7^ substitutions per site per year (95% HPD 4.4–7.0×10^−7^) under the BICEPS model and 5.0×10^−7^ substitutions per site per year (95% HPD, 3.4–6.5×10^−7^) under the MASCOT model. The credible intervals largely overlap, and, again, the estimate based on the BICEPS model is preferred here. The substitution rate estimates are somewhat higher than previously published estimates; for example, Deng *et al.* inferred a substitution rate of 2.2×10^−7^ [67]. The disparity between estimates can be explained by the commonly observed phenomenon described by Duchêne *et al.* [81] in which rates for outbreaks are higher than those for which samples have been obtained over a longer period. This is because substitution rates measured over a brief period contain deleterious mutations not yet removed by either natural selection or substitutional saturation [81].

### Population size estimates show rapid growth in the outbreak and rapid decline following formal intervention

A single recent common ancestor followed by divergence and increased population size implies a recent point source introduction of SE-19C01 into NZ. The BICEPS model shows an exponential increase in the effective population size between mid-2019 and mid-2021 that coincided with the increase in clinical human cases with multiple point source outbreaks [12]. This increase was most likely attributable to amplification of the strain within the poultry industry, as has been seen in other outbreaks [67,82]. A sharp decrease in SE-19C01 effective population size (from a mean of 10.23 to 1.48) is inferred with a mean date of 20 May 2021, coinciding closely with the formal response by MPI and MoH which commenced on 22 April 2021 [6]. The response included depopulating flocks where the outbreak strain was detected and public health messaging advising how consumers could protect themselves against *S*. Enteritidis, such as by cooking eggs thoroughly. The timing and size of the inferred drop in effective population size, along with the observed drop in human cases from 12 cases in April 2021 to 4 cases in May 2021 [12], provide strong evidence that the formal response had an immediate and significant effect in curbing the outbreak.

### Hierarchical clustering and phylogenetics support three major clades

Hierarchical clustering supports three clades, with clade 1 containing only isolates from clinical human cases in the Auckland region and isolates sampled from egg producer B. Clade 1 has isolates from clinical cases collected between 23 May 2019 and 2 June 2021. This clade includes isolates from a large point source outbreak in 2019 of 17 cases linked to a raw egg dessert from an Auckland restaurant. Egg producer B supplied eggs to the restaurant [12]. Clade 2 includes clinical isolates, with the majority (39/42) collected between 23 April 2020 and 6 July 2021, that come from the central North Island and include poultry isolates from all sampled egg producers except producer B, all broiler producers and hatchery A. Clade 3 is composed of clinical human isolates sampled after the formal intervention, collected between 18 November 2021 and 3 June 2022, from the mid-North Island as well as the South Island. It also included poultry isolates sampled at hatchery A and broiler producers F and G.

The composition of the three clades suggests a shift in the human case locations from the upper North Island of NZ to the lower North Island and eventually the South Island. The early stage of this outbreak in 2019 and 2020 was mainly egg-related (Fig. 3, clade 1). Later in the outbreak (late 2020/early 2021), there is evidence of infection at both egg and broiler producers (Fig. 3, clade 2). As industry interventions took effect, infection shifted to broiler producers only (Fig. 3, clade 3). The reason for this shift remained unclear to us, but it may be insightful to industry and public health agencies with a greater understanding of the underlying factors. The under-representation of human clinical cases in clade 2 and clade 3 may potentially be due to chicken meat almost always being consumed well-cooked; however, eggs may be consumed raw or partially cooked. The over-representation of the hatchery A in clade 3 may be due to the sampling strategy guided by the ‘working solution’ held during the formal response [12].

### Hatchery A is a significant source in later stages of the outbreak, with other transmission pathways being more cryptic

The topology of the inferred phylogenies and inferred migration rates indicates that hatchery A played a role in the transmission of the strain throughout the poultry production systems. However, it is not possible to directly infer transmission events between producers (including hatchery A) based on the CCD tree alone. The MASCOT model indicates that hatchery A is the most probable root location of the outbreak, although with low posterior probability. It further indicates several transmissions to egg supplier B during the early part of the outbreak, again with low posterior probability. This period coincides with a major cluster in October 2019 involving 17 cases associated with a raw egg dessert served in an Auckland restaurant. The more distant internal tree nodes are from sampled isolates, the more uncertainty there is in MASCOT’s inferences of their location, hence the low probabilities associated with inferred root location [27]. b_migration values do not support a lot of transmission from hatchery A to egg producer B. Therefore, stronger evidence would be required to demonstrate that hatchery A was the root.

A transmission event with low probability (0.28) to an uncertain intermediary mid to late 2020 was followed by transmission from the intermediate to egg producers C and E and broiler producers D, F and G. The most probable intermediaries were inferred to be hatchery A or broiler producer F. For the same period, sporadic transmission was inferred between hatchery A and broiler producer D. Transmission from hatchery A to broiler producer D is supported by high b_migration rates between the demes. This period coincided with the first detection of *S*. Enteritidis in the NZ poultry production environment. Furthermore, transmission from hatchery A to broiler producer D is supported by the initial traceback investigation that found that broiler producer D and a rearer company for various implicated egg layer flocks were supplied by hatchery A on the same day (19 January 2021) [6]. Isolates from the rearer company were not available for this study. However, this evidence suggests that diversification could have occurred before distribution to the various poultry producers, including egg producers C, E and H.

While individual transmission events can be hard to determine, the conditional clade distribution tree strongly supports transmission from hatchery A to broiler producers F and G, post-mid-2021, following the formal response. This pattern of transmission is also supported by the b_migration rates between demes. Even though transmission from hatchery A to producers B, D, E, F and G is unexpected due to a more diverse population seeded by a less diverse population, depopulation of infected flocks during and after the initial investigation (especially hatchery A) would have impacted diversity by introducing various population bottlenecks.

### Limitations of using MASCOT

Whilst b_migration rates between demes strongly support transmission from hatchery A to the human population, this demonstrates a limitation of MASCOT analyses as hatchery A does not supply consumers directly. MASCOT assumes a fixed number of demes through time, with all of them sampled, fixed population sizes within demes and constant migration rates through time. All these assumptions are violated to varying extents by the reality of this outbreak. For example, rearer farms that supply layer flocks after raising day-old chicks to laying age were not included. A farm being completely depopulated is equivalent to a deme disappearing. Migration rates may change through time for several reasons, but most obviously at the time of formal interventions to prevent transmission. Population size is far from constant within demes, especially within a single source outbreak. The modelled population size here is a rough proxy for the number of infected animals (or humans) within a deme. A recent extension to MASCOT, allowing variable population sizes through time, was recently released [83]. However, attempts to get results from the new model with this relatively sparse dataset, consisting of highly similar and therefore relatively uninformative sequences, were not successful.

The inferred effective population sizes all have broad, overlapping distributions. They are similar for producers B, D, E, G, H and humans, while hatchery A and producers C and F have a much lower mean and distribution (Fig. S2). These broad estimates are strongly affected by the sampling protocols. For this study, clinical human samples were collected between May 2019 and June 2022; while a single poultry sample was collected in June 2020, the remainder of the poultry samples were only collected from February 2021 onwards. In addition, only poultry facilities that yielded positive samples were included and human isolates are limited to those cases that sought medical advice. In addition, poultry flocks that were depopulated due to the intervention introduced by MPI had a direct impact on the effective bacterial population size.

When comparing estimated dated phylogenies and rates between MASCOT and BICEPS, we note that BICEPS is also a mis-specified model here as the population structure is not modelled. However, the flexibility of the BICEPS model, due to the flexibility of the non-parametric population size function compared to the rigidity of the MASCOT model, means that divergence dates and substitution rates estimated under BICEPS are to be preferred.

### Multiple modes of transmission and vectors are known for *Salmonella*

This study supports the working solution at the time of the formal response of MPI and MoH that a hatchery that supplied day-old chicks to the industry was the source of infection in downstream operations directly and indirectly, with strong support during the latter part of the outbreak. However, other avenues of transmission cannot be discounted. These include poultry feed, birds, rodents, insects, staff, modes of transport and equipment, to name a few. *Salmonella* contamination of poultry feed is well documented [84,86]. *S*. Typhimurium has been described as causing septicaemia in wild bird populations in NZ, as well as posing a significant zoonotic risk [87]. A human outbreak of an *S*. Typhimurium between 2020 and 2021 in the USA was linked to wild songbirds [88]. Olga Obukhovska found that migrating wild birds can carry *S*. Enteritidis over long distances and are a threat to commercial poultry flocks and humans [89]. A persistent common-source multicounty outbreak of *S*. Enteriditis ST11 PT8 between 2011 and 2016, associated with feeder mice, occurred in Europe. The implicated feeder mice were produced on a rodent farm in Lithuania and marketed across Europe and the UK as feed for pet reptiles [90,91]. In our study, isolates from two hedgehogs trapped in the vicinity of hatchery A were indistinguishable (0 core SNP difference) from isolates taken from hatchery A and broiler producers F and G. However, hedgehogs are not a migratory species [92]. Even though the home range for hedgehogs varies amongst habitat types [93], a NZ study estimated a mean home range size of 45.3 ha for males and 12.7 ha for females [94]. Therefore, the distances between hatchery A and broiler producers F and G (over 23 km) are too great to reasonably consider hedgehogs as a vector over such a brief period.

The role of poultry ectoparasites in *Salmonella* transmission in this outbreak cannot be excluded. Studies have shown that the ectoparasite *Dermanyssus* gallinae (red mite) acts as a vector for *S*. Gallinarum [95]. Not only has it been shown that *D. gallinae* transmits *Salmonella* to poultry, but that *D. gallinae* protects the pathogen from adverse environmental conditions such as dehydration, excessive levels of ammonium compounds, lack of nutrients and even contact with antimicrobial substances. Valiente *et al.* have shown that *D. gallinae* may act as a vector for *S*. Enteritidis under experimental conditions [96].

## Conclusion

The results presented here are consistent with traditional investigative evidence but provide additional detailed insight into transmission dynamics. This study provided evidence that outbreak strain SE-19C01 was introduced into the NZ poultry industry just months prior to the first identified human isolate, followed by multiple transmission and amplification events within the poultry industry. The exponential increase in the SE-19C01 population size within the poultry industry coincided with the increase in notified clinical human cases, as well as with multiple point source outbreaks. Furthermore, this study showed that interventions introduced by the NZ MoH, MPI and the poultry industry coincided with a large drop in the effective bacterial population size, suggesting that the control measures introduced were successful.

## Supplementary material

10.1099/mgen.0.001525 Uncited Supplementary Material 1.

## Abbreviations

BICEPS: Bayesian integrated coalescent epoch plots
CCD: conditional clade distribution
cgMLST: core genome multilocus sequence typing
DIC: deviance information criterion
ERL: Enteric Reference Laboratory
ESR: Institute of Environmental Science and Research
95% HPD: 95% highest posterior density
MCC: maximum clade credibility
MCMC: Markov chain Monte Carlo
ML: maximum likelihood
MLST: multilocus sequence typing
mMDS: metric multidimensional scaling
MoH: Ministry of Health
MPI: Ministry for Primary Industries
MRCA: most recent common ancestor
NZ: New Zealand
PT8: phage type 8
*S.* Enteritidis: *Salmonella enterica* subs. *enterica* serovar Enteritidis
ST: sequence type
