# Corrigendum: Temporal reconstruction of a Salmonella enteritidis ST11 outbreak in New Zealand

**DOI:** 10.1099/mgen.0.001594

**Published:** 2025-12-04

**Authors:** Hugo Strydom, Jackie Wright, Collette Bromhead, David Welch, Ernest Williams, Kerry Mulqueen, Joep de Ligt, Patrick J. Biggs, Shevaun Paine, Sarah Jefferies, Nigel French

**Affiliations:** 1 Enteric Reference Laboratory, Institute of Environmental Science and Research, Upper Hutt, New Zealand; 2 Tāwharau Ora | School of Veterinary Science, Massey University, Palmerston North, New Zealand; 3 Health and Environment Group, Institute of Environmental Science and Research, Christchurch Science Centre, Christchurch, New Zealand; 4 School of Health Sciences, Massey University, Wellington, New Zealand; 5 School of Computer Science, University of Auckland, Auckland, New Zealand; 6 Poultry Industry Association of New Zealand (PIANZ), Auckland, New Zealand; 7 Genomics and Bioinformatics, Health and Environment Group, Institute of Environmental Science and Research, Porirua, New Zealand; 8 School of Food Technology and Natural Sciences, Massey University, Palmerston North, New Zealand; 9 New Zealand Food Safety Science and Research Centre, Massey University, Palmerston North, New Zealand; 10 ESR Health Intelligence and Surveillance, Health and Environment Group, Institute of Environmental Science and Research, Porirua, New Zealand

**Keywords:** Bayesian integrated coalescent epoch plots (BICEPS), New Zealand, Salmonella Enteritidis, marginal approximation of the structured coalescent (MASCOT), outbreak, poultry

The authors would like to correct the acknowledgement statement included in the version of record of this article to better reflect the contribution of their colleague Jing Wang.

The correct acknowledgement statement should read:

We would like to thank MPI, MoH and PIANZ for providing bacterial cultures and making sequencing data available for analysis. We would like to thank Roger Cook for his ongoing advice and support. We acknowledge that the Ministry of Health funds ESR to run national surveillance of human salmonellosis, outbreak investigation and typing of clinical samples used in this study. We would also like to acknowledge the Health Intelligence and Surveillance Group at ESR for leadership in national outbreak detection and investigation. Thanks also to all Public Health Service teams, especially the Auckland Regional Public Health Service, who were key in the outbreak investigation. We would also like to acknowledge Penelope Hancock from the Enteric Reference Laboratory at ESR for subtyping *Salmonella* isolates into serovars, and members of the ESR sequencing facility team for the sequencing of isolates. We would like to acknowledge Jing Wang for her work of developing and implementing a routine *Salmonella* identification and clustering pipeline that enabled the recognition and identification of *S*. Enteritidis_2019_C_01, as well as for her contributions in the subsequent outbreak investigation. We would like to express our gratitude to both internal and external reviewers of this manuscript.

The acknowledgement statement in the original text will be updated to reflect this change. The authors apologise for any inconvenience caused.

