# Supplementary material for: Temporal reconstruction of a Salmonella Enteritidis ST11 outbreak in New Zealand
Source: Microb Genom. 2025 Oct 30;11(10):001525. doi: 10.1099/mgen.0.001525 (PMC12574974; doi:10.1099/mgen.0.001525)
Supplement: Uncited Supplementary Material 1. [file mgen-11-01525-s001.pdf]

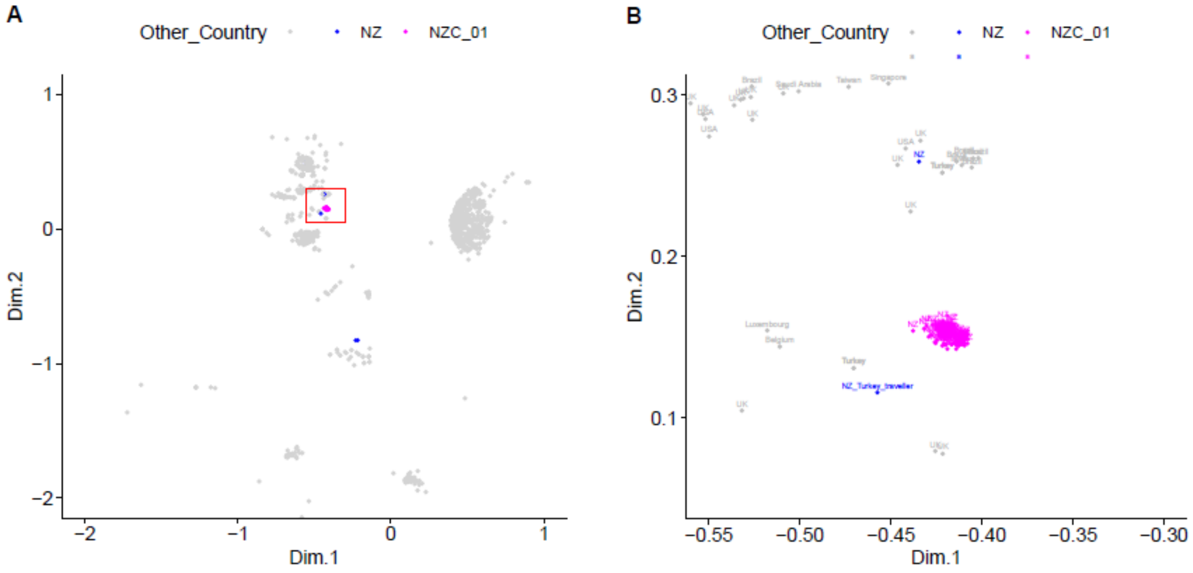

Figure S1 A metric multi-dimensional scaling of the cgMLST allele profile distance matrix for 1476 *S.* Enteritidis ST11 genomes from Enterobase.

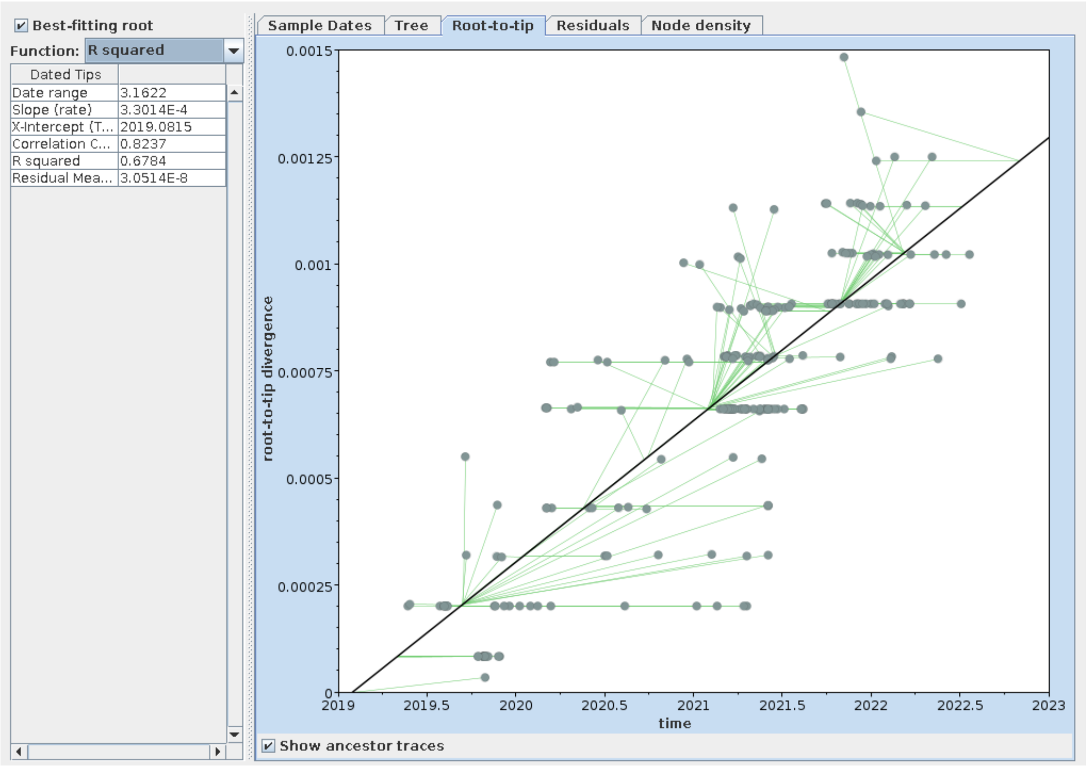

Figure S2 A linear regression of root-to-tip genetic distance (core SNP) against sampling dates generated using TempEST.

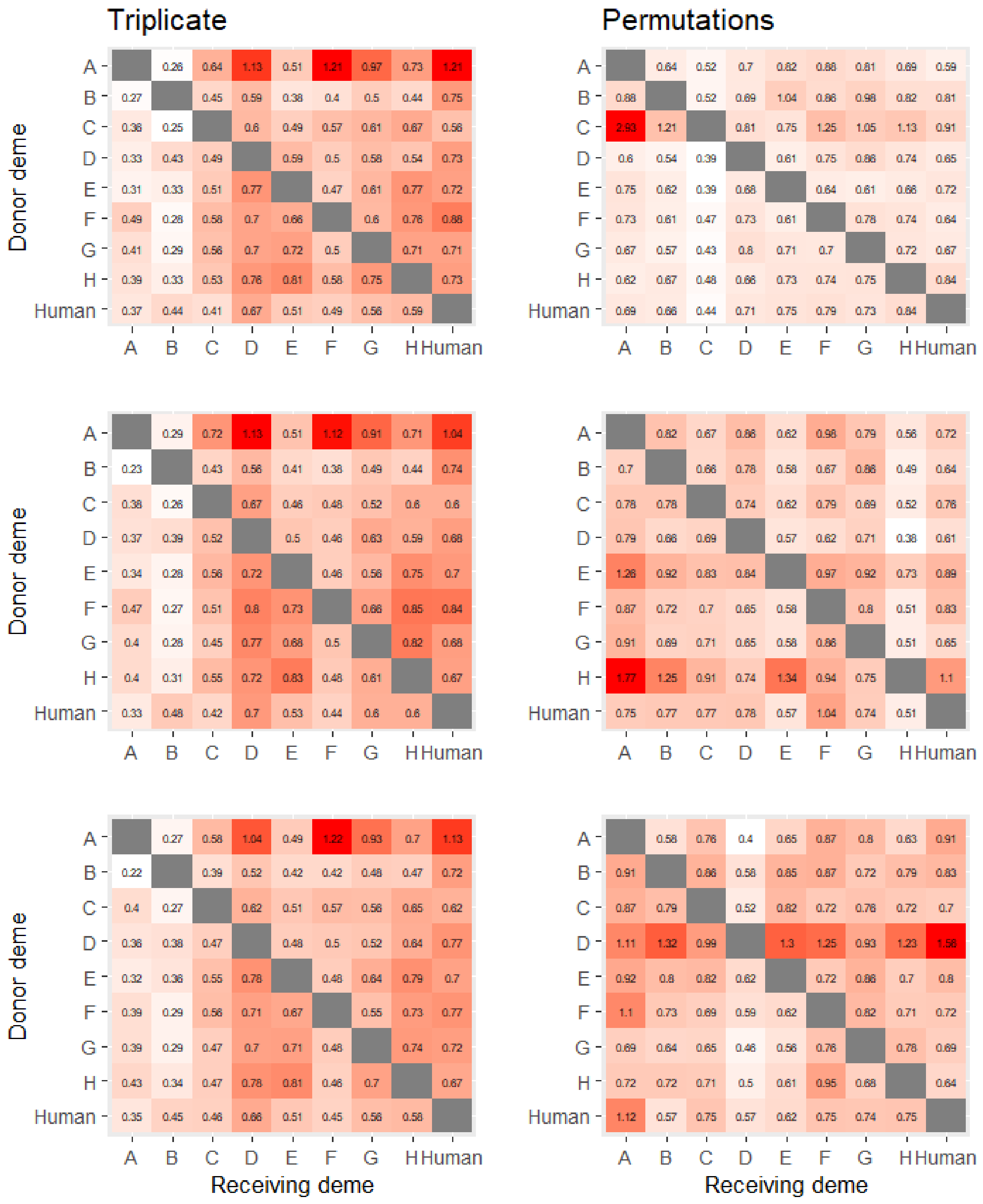

Figure S3 Matrix showing b_migration (x-axis) to (y-axis) obtained using the MASCOT model. The three figures on the left are from triplicate analysis, while the three figures on the right are from permutated analysis.

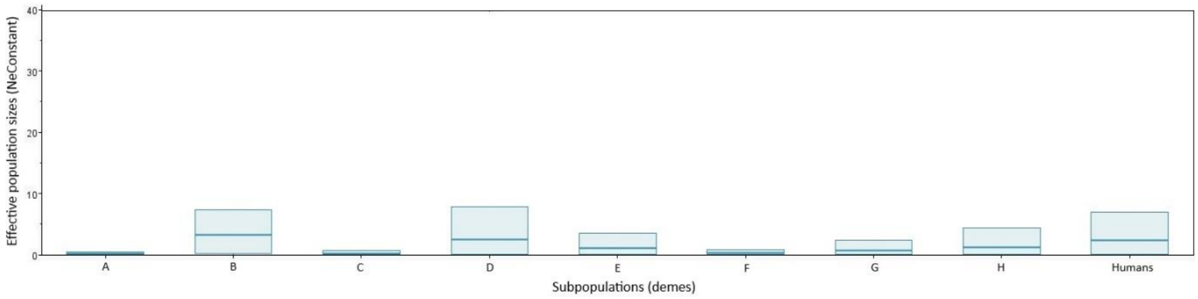

Figure S4. Box and whiskers plot showing the median and 95% high posterior density interval of the effective bacterial population (NeConstant) for poultry producers A to H and humans, inferred using MASCOT.

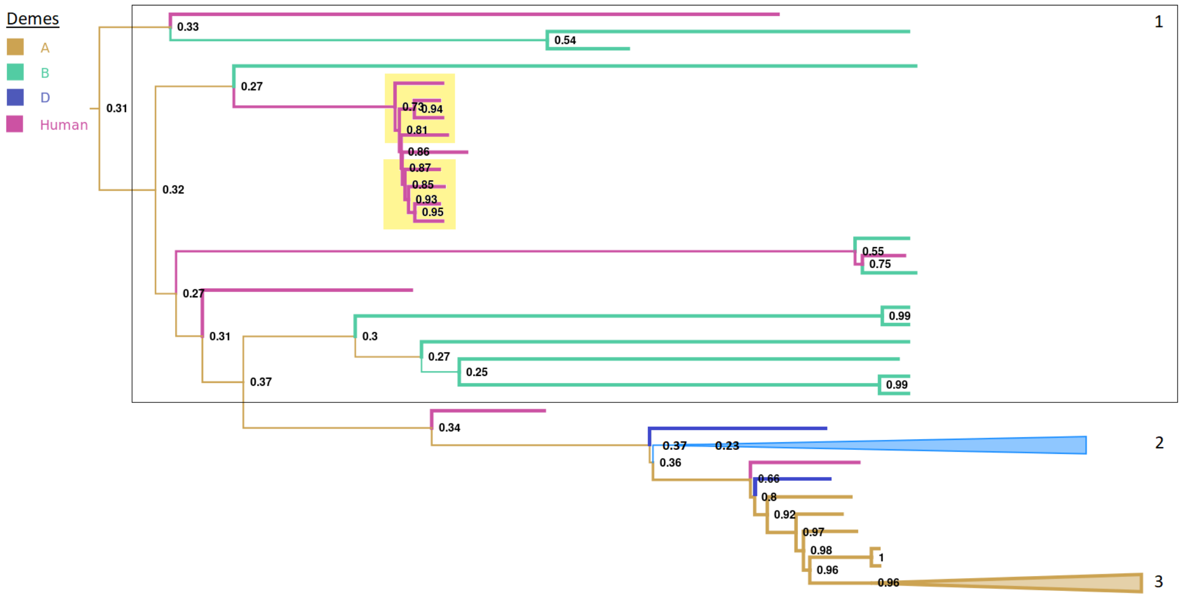

Figure S5 Time-scaled phylogeny using approximated structured modelling (MASCOT) of *S*. Enteritidis SE-19C01 isolates obtained from implicated poultry producers, 10 randomly selected clinical human isolates as well as 8 clinical isolates epidemiologically linked to the 2019 Auckland restaurant cluster (indicated in yellow squares). Phylogeny branches are coloured according to their most probable ancestral state of sister branches. Nodes are labelled with the maximum posterior probability of the ancestral state.

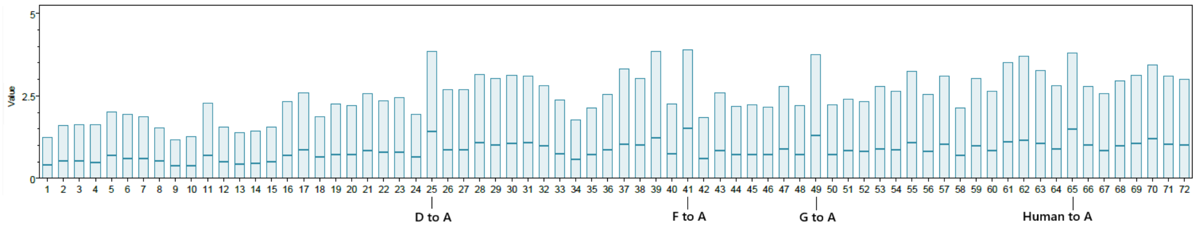

Figure S6 Box plot showing the mean value and 95% high posterior density for b_migration obtained using MASCOT.

Table S1 Euclidean distances (km) between poultry producer sites from which isolates were indistinguishable (0 SNPs) from hedgehog isolates. Producers F and G operate from multiple sites.

	A	A	F3	G1	G2	G5
**A**	0.00	0.79	354.77	28.69	63.40	23.64
**A**	0.79	0.00	353.99	28.79	64.04	24.38
**F3**	354.77	353.99	0.00	353.86	412.98	378.02
**G1**	28.69	28.79	353.86	0.00	81.42	41.58
**G2**	63.40	64.04	412.98	81.42	0.00	40.96
**G5**	23.64	24.38	378.02	41.58	40.96	0.00

Table S2 Short read sequences (SRA) for all isolates included in this study

Sample ID	Collection date	Host	Region	Environmental source	Poultry producers	BioSample	SRA
**19ER2117**	23/05/2019	Human	Waitemata	-	-	SAMN19844825	SRR14917104
**19ER2161**	27/05/2019	Human	Waitemata	-	-	SAMN19844826	SRR14917103
**19ER2926**	28/07/2019	Human	Auckland	-	-	SAMN19842063	SRR14902908
**19ER2978**	6/08/2019	Human	Manukau	-	-	SAMN19842064	SRR14902943
**19ER2979**	6/08/2019	Human	Manukau	-	-	SAMN19842065	SRR14902942
**19ER2984**	6/08/2019	Human	Waikato	-	-	SAMN19844827	SRR14917092
**19ER3036**	9/08/2019	Human	Manukau	-	-	SAMN19844828	SRR14917054
**19ER3102**	12/08/2019	Human	Manukau	-	-	SAMN19844829	SRR14917087
**19ER3149**	12/08/2019	Human	Auckland	-	-	SAMN46989324	SRR32474046
**19ER3852**	18/09/2019	Human	Auckland	-	-	SAMN19844830	SRR14917074
**19ER3941**	20/09/2019	Human	Waikato	-	-	SAMN19844831	SRR14917079
**19ER4295**	15/10/2019	Human	Hutt Valley	-	-	SAMN19844833	SRR14917085
**19ER4296**	14/10/2019	Human	Wellington	-	-	SAMN19844832	SRR14917080
**19ER4435**	24/10/2019	Human	Wairarapa	-	-	SAMN19844834	SRR14917084
**19ER4494**	25/10/2019	Human	Waitemata	-	-	SAMN19844837	SRR14917100
**19ER4495**	25/10/2019	Human	Waitemata	-	-	SAMN19844836	SRR14917101
**19ER4496**	25/10/2019	Human	Waitemata	-	-	SAMN19844835	SRR14917102
**19ER4553**	29/10/2019	Human	Auckland	-	-	SAMN19844838	SRR14917099
**19ER4554**	29/10/2019	Human	Northland	-	-	SAMN19844839	SRR14917098
**19ER4555**	29/10/2019	Human	Manukau	-	-	SAMN19844840	SRR14917097
**19ER4557**	31/10/2019	Human	Manukau	-	-	SAMN19844841	SRR14917096
**19ER4570**	1/11/2019	Human	Waitemata	-	-	SAMN19844842	SRR14917095
**19ER4583**	4/11/2019	Human	Manukau	-	-	SAMN19844843	SRR14917094
**19ER4783**	19/11/2019	Human	Auckland	-	-	SAMN19844845	SRR14917091
**19ER4798**	17/11/2019	Human	Auckland	-	-	SAMN19844844	SRR14917093
**19ER4831**	22/11/2019	Human	Manukau	-	-	SAMN19844846	SRR14917090
**19ER4835**	25/11/2019	Human	Auckland	-	-	SAMN19844848	SRR14917088
**19ER4864**	28/11/2019	Human	Auckland	-	-	SAMN19844849	SRR14917051
**19ER4867**	23/11/2019	Human	Waitemata	-	-	SAMN19844847	SRR14917089
**19ER4888**	2/12/2019	Human	Waitemata	-	-	SAMN19844850	SRR14917052
**19ER4968**	7/12/2019	Human	Waitemata	-	-	SAMN19844851	SRR14917053
**20ER0066**	18/12/2019	Human	Waitemata	-	-	SAMN19844852	SRR14917081
**20ER0235**	8/01/2020	Human	Waikato	-	-	SAMN19844853	SRR14917082
**20ER0489**	30/01/2020	Human	Manukau	-	-	SAMN19844854	SRR14917083
**20ER0635**	14/02/2020	Human	Waitemata	-	-	SAMN19844855	SRR14917055
**20ER0889**	5/03/2020	Human	Waitemata	-	-	SAMN19844860	SRR14917060
**20ER0895**	3/03/2020	Human	Waitemata	-	-	SAMN19844858	SRR14917058
**20ER0896**	2/03/2020	Human	Auckland	-	-	SAMN19844857	SRR14917057
**20ER0899**	4/03/2020	Human	Waitemata	-	-	SAMN19844859	SRR14917059
**20ER0941**	1/03/2020	Human	Auckland	-	-	SAMN19844856	SRR14917056
**20ER1002**	11/03/2020	Human	Manukau	-	-	SAMN19844861	SRR14917061

Sample ID	Collection date	Host	Region	Environmental source	Poultry producers	BioSample	SRA
**20ER1008**	12/03/2020	Human	Waitemata	-	-	SAMN19844862	SRR14917062
**20ER1095**	14/03/2020	Human	Waitemata	-	-	SAMN19844863	SRR14917063
**20ER1096**	19/03/2020	Human	Waitemata	-	-	SAMN19844864	SRR14917064
**20ER1287**	23/04/2020	Human	Auckland	-	-	SAMN19844865	SRR14917086
**20ER1360**	6/05/2020	Human	Auckland	-	-	SAMN19844866	SRR14917065
**20ER1598**	30/05/2020	Human	Wellington	-	-	SAMN19844867	SRR14917066
**20ER1624**	5/06/2020	Human	Canterbury	-	-	SAMN19844868	SRR14917067
**20ER1865**	1/07/2020	Human	Manukau	-	-	SAMN19844869	SRR14917068
**20ER1933**	6/07/2020	Human	Manukau	-	-	SAMN19844870	SRR14917069
**20ER1948**	6/07/2020	Human	Waitemata	-	-	SAMN19844871	SRR14917070
**20ER2166**	29/07/2020	Human	Manukau	-	-	SAMN19844872	SRR14917071
**20ER2271**	4/08/2020	Human	Waitemata	-	-	SAMN19844873	SRR14917072
**20ER2429**	11/08/2020	Human	Waitemata	-	-	SAMN46989325	SRR32474045
**20ER2569**	18/08/2020	Human	Manukau	-	-	SAMN19844874	SRR14917073
**20ER3173**	25/09/2020	Human	Bay of Plenty	-	-	SAMN19844875	SRR14917075
**20ER3519**	19/10/2020	Human	Waitemata	-	-	SAMN19844876	SRR14917076
**20ER3560**	25/10/2020	Human	Waitemata	-	-	SAMN19844877	SRR14917077
**20ER3646**	2/11/2020	Human	Manukau	-	-	SAMN19844878	SRR14917078
**20ER4089**	10/12/2020	Human	Waitemata	-	-	SAMN46989326	SRR32474034
**21ER0015**	17/12/2020	Human	Waitemata	-	-	SAMN19066278	SRR14716988
**21ER0114**	6/01/2021	Human	Auckland	-	-	SAMN19066291	SRR14717038
**21ER0222**	12/01/2021	Human	Hutt Valley	-	-	SAMN19066318	SRR14716895
**21ER0250**	21/12/2020	Human	Manukau	-	-	SAMN19066322	SRR14716896
**21ER0477**	6/02/2021	Human	Waitemata	-	-	SAMN19066360	SRR14716931
**21ER0614**	17/02/2021	Human	Waitemata	-	-	SAMN19066386	SRR14717091
**21ER0622**	17/02/2021	Poultry	-	Broiler Producer	D	SAMN46989004	SRR32473976
**21ER0702**	23/02/2021	Human	Manukau	-	-	SAMN19066400	SRR14717076
**21ER0726**	24/02/2021	Poultry	-	Broiler Producer	D	SAMN46989005	SRR32473975
**21ER0758**	1/03/2021	Human	Waikato	-	-	SAMN19066401	SRR14717075
**21ER0779**	3/03/2021	Poultry	-	Broiler Producer	D	SAMN46989006	SRR32473964
**21ER0782**	5/03/2021	Human	Waitemata	-	-	SAMN19066405	SRR14717070
**21ER0784**	5/03/2021	Human	Waitemata	-	-	SAMN19066406	SRR14717069
**21ER0803**	5/03/2021	Human	Hawke's Bay	-	-	SAMN18642333	SRR14160460
**21ER0829**	12/03/2021	Human	Waikato	-	-	SAMN19066415	SRR14717059
**21ER0836**	11/03/2021	Poultry	-	Hatchery	A	SAMN46989007	SRR32473953
**21ER0874**	8/03/2021	Human	Auckland	-	-	SAMN19066418	SRR14717056
**21ER0875**	8/03/2021	Human	Auckland	-	-	SAMN19066419	SRR14717055
**21ER0881**	11/03/2021	Human	Waitemata	-	-	SAMN19066424	SRR14717050
**21ER0906**	16/03/2021	Bovine	Manawatu	Cow	-	SAMN46989350	SRR32474023
**21ER0913**	14/03/2021	Human	Taupo/Rotorua	-	-	SAMN19066425	SRR14717048
**21ER0916**	17/03/2021	Human	Auckland	-	-	SAMN19066427	SRR14717046
**21ER0920**	15/03/2021	Human	South Canterbury	-	-	SAMN19066430	SRR14717043
**21ER0958**	16/03/2021	Human	Auckland	-	-	SAMN19066431	SRR14717042
**21ER0981**	19/03/2021	Human	Auckland	-	-	SAMN19066436	SRR14717036
**21ER0984**	11/03/2021	Poultry	-	Hatchery	A	SAMN46989008	SRR32473942
**21ER1007**	24/03/2021	Human	Manawatu	-	-	SAMN19066443	SRR14717029
**21ER1014**	21/03/2021	Human	Waitemata	-	-	SAMN19066445	SRR14717026
**21ER1016**	20/03/2021	Human	Manukau	-	-	SAMN19066447	SRR14717024
**21ER1060**	22/03/2021	Human	Auckland	-	-	SAMN19066448	SRR14717023
**21ER1062**	21/03/2021	Human	Manukau	-	-	SAMN19066449	SRR14717022
**21ER1064**	25/03/2021	Feline	Auckland	Cat	-	SAMN46989355	SRR32474019
**21ER1065**	25/03/2021	Canine	Auckland	Dog	-	SAMN46989351	SRR32474018
**21ER1069**	22/03/2021	Poultry	-	Hatchery	A	SAMN46989009	SRR32473931
**21ER1135**	1/04/2021	Human	Manukau	-	-	SAMN19066456	SRR14717014
**21ER1158**	29/03/2021	Poultry	-	Hatchery	A	SAMN46989010	SRR32473920
**21ER1159**	24/03/2021	Poultry	-	Egg Producer	H	SAMN46989011	SRR32473909
**21ER1225**	7/04/2021	Human	Taranaki	-	-	SAMN19066479	SRR14716999
**21ER1234**	6/04/2021	Poultry	-	Hatchery	A	SAMN46989012	SRR32473898
**21ER1291**	5/04/2021	Human	Waikato	-	-	SAMN19066485	SRR14716957
**21ER1299**	13/04/2021	Human	Manukau	-	-	SAMN19066491	SRR14716960
**21ER1338**	15/04/2021	Feline	Wairarapa	Cat	-	SAMN46989356	SRR32474017
**21ER1339**	13/04/2021	Poultry	-	Egg Producer	H	SAMN46989013	SRR32473887
**21ER1379**	17/04/2021	Human	Waikato	-	-	SAMN19842072	SRR14902935
**21ER1385**	22/04/2021	Human	Hawke's Bay	-	-	SAMN19842081	SRR14902925
**21ER1388**	18/04/2021	Human	Hutt Valley	-	-	SAMN19842073	SRR14902933

Sample ID	Collection date	Host	Region	Environmental source	Poultry producers	BioSample	SRA
**21ER1389**	16/04/2021	Human	Waikato	-	-	SAMN19842071	SRR14902936
**21ER1413**	22/04/2021	Human	Northland	-	-	SAMN19842080	SRR14902926
**21ER1419**	19/04/2021	Human	Manukau	-	-	SAMN19842076	SRR14902930
**21ER1422**	19/04/2021	Human	Waitemata	-	-	SAMN19842077	SRR14902929
**21ER1427**	9/04/2021	Poultry	-	Egg Producer	H	SAMN46989014	SRR32473974
**21ER1448**	9/04/2021	Poultry	-	Egg Producer	H	SAMN46989015	SRR32473973
**21ER1451**	19/04/2021	Poultry	-	Hatchery	A	SAMN46989016	SRR32473972
**21ER1457**	26/04/2021	Human	Manukau	-	-	SAMN19842084	SRR14902921
**21ER1523**	26/04/2021	Poultry	-	Hatchery	A	SAMN46989017	SRR32473971
**21ER1532**	3/05/2021	Human	Waikato	-	-	SAMN19842093	SRR14902906
**21ER1537**	27/04/2021	Poultry	-	Hatchery	A	SAMN46989018	SRR32473970
**21ER1538**	3/05/2021	Poultry	-	Hatchery	A	SAMN46989019	SRR32473969
**21ER1603**	10/05/2021	Caprine	Manawatu	Goat	-	SAMN46989352	SRR32474016
**21ER1632**	10/05/2021	Poultry	-	Hatchery	A	SAMN46989020	SRR32473968
**21ER1712**	17/05/2021	Human	Wellington	-	-	SAMN19842114	SRR14902883
**21ER1747**	16/05/2021	Poultry	-	Egg Producer	E	SAMN46989021	SRR32473967
**21ER1748**	16/05/2021	Poultry	-	Egg Producer	E	SAMN46989022	SRR32473966
**21ER1749**	17/05/2021	Poultry	-	Egg Producer	C	SAMN46989023	SRR32473965
**21ER1750**	17/05/2021	Poultry	-	Egg Producer	C	SAMN46989024	SRR32473963
**21ER1794**	14/05/2021	Poultry	-	Broiler Producer	G	SAMN46989025	SRR32473962
**21ER1827**	25/05/2021	Poultry	-	Egg Producer	E	SAMN46989026	SRR32473961
**21ER1862**	28/05/2021	Human	Manukau	-	-	SAMN19842045	SRR14902907
**21ER1863**	28/05/2021	Human	Manukau	-	-	SAMN19842047	SRR14902923
**21ER1879**	15/05/2021	Human	Waikato	-	-	SAMN19842113	SRR14902884
**21ER1883**	26/05/2021	Poultry	-	Hatchery	A	SAMN46989027	SRR32473960
**21ER1909**	2/06/2021	Human	Waitemata	-	-	SAMN19842127	SRR14902869
**21ER1920**	20/05/2021	Poultry	-	Egg Producer	B	SAMN46989028	SRR32473959
**21ER1921**	1/06/2021	Poultry	-	Egg Producer	C	SAMN46989029	SRR32473958
**21ER1994**	1/06/2021	Poultry	-	Egg Producer	E	SAMN46989030	SRR32473957
**21ER1995**	1/06/2021	Poultry	-	Egg Producer	E	SAMN46989031	SRR32473956
**21ER1996**	1/06/2021	Poultry	-	Egg Producer	E	SAMN46989032	SRR32473955
**21ER2002**	2/06/2021	Poultry	-	Egg Producer	B	SAMN46989033	SRR32473954
**21ER2003**	2/06/2021	Poultry	-	Egg Producer	B	SAMN46989034	SRR32473952
**21ER2004**	2/06/2021	Poultry	-	Egg Producer	B	SAMN46989035	SRR32473951
**21ER2005**	2/06/2021	Poultry	-	Egg Producer	B	SAMN46989036	SRR32473950
**21ER2006**	2/06/2021	Poultry	-	Egg Producer	B	SAMN46989037	SRR32473949
**21ER2007**	2/06/2021	Poultry	-	Egg Producer	B	SAMN46989038	SRR32473948
**21ER2008**	2/06/2021	Poultry	-	Egg Producer	B	SAMN46989039	SRR32473947
**21ER2009**	2/06/2021	Poultry	-	Egg Producer	C	SAMN46989040	SRR32473946
**21ER2026**	11/06/2021	Poultry	-	Egg Producer	B	SAMN46989041	SRR32473945
**21ER2027**	11/06/2021	Poultry	-	Egg Producer	B	SAMN46989042	SRR32473944
**21ER2045**	14/06/2021	Human	Tairawhiti	-	-	SAMN46989327	SRR32474015
**21ER2048**	17/06/2020	Poultry	-	Egg Producer	B	SAMN46989043	SRR32473943
**21ER2050**	8/06/2021	Poultry	-	Egg Producer	E	SAMN46989044	SRR32473941
**21ER2062**	15/06/2021	Human	Southern	-	-	SAMN46989328	SRR32474014
**21ER2063**	14/06/2021	Human	Hawke's Bay	-	-	SAMN46989329	SRR32474044
**21ER2066**	14/06/2021	Human	Taranaki	-	-	SAMN46989330	SRR32474043
**21ER2166**	21/06/2021	Human	Waikato	-	-	SAMN46989331	SRR32474042
**21ER2171**	11/06/2021	Poultry	-	Broiler Producer	F	SAMN46989045	SRR32473940
**21ER2187**	22/06/2021	Human	Hawke's Bay	-	-	SAMN46989332	SRR32474041
**21ER2328**	5/07/2021	Poultry	-	Broiler Producer	G	SAMN46989046	SRR32473939
**21ER2352**	6/07/2021	Human	Waitemata	-	-	SAMN46989333	SRR32474040
**21ER2490**	15/07/2021	Poultry	-	Egg Producer	C	SAMN46989047	SRR32473938
**21ER2578**	16/07/2021	Poultry	-	Broiler Producer	G	SAMN46989048	SRR32473937
**21ER2579**	20/07/2021	Poultry	-	Hatchery	A	SAMN46989049	SRR32473936
**21ER2945**	9/08/2021	Poultry	-	Egg Producer	H	SAMN46989050	SRR32473935
**21ER2946**	9/08/2021	Poultry	-	Egg Producer	H	SAMN46989051	SRR32473934
**21ER3048**	12/08/2021	Poultry	-	Unknown	-	SAMN46989052	SRR32473933
**21ER3053**	12/08/2021	Poultry	-	Egg Producer	E	SAMN46989053	SRR32473932
**21ER3054**	12/08/2021	Poultry	-	Egg Producer	E	SAMN46989054	SRR32473930
**21ER3055**	12/08/2021	Poultry	-	Egg Producer	E	SAMN46989055	SRR32473929
**21ER3537**	27/09/2021	Poultry	-	Hatchery	A	SAMN46989056	SRR32473928
**21ER3630**	3/10/2021	Poultry	-	Hatchery	A	SAMN46989057	SRR32473927
**21ER3631**	4/10/2021	Poultry	-	Broiler Producer	G	SAMN46989058	SRR32473926
**21ER3633**	1/10/2021	Poultry	-	Broiler Producer	F	SAMN46989059	SRR32473925

Sample ID	Collection date	Host	Region	Environmental source	Poultry producers	BioSample	SRA
**21ER3757**	11/10/2021	Poultry	-	Broiler Producer	F	SAMN46989060	SRR32473924
**21ER3814**	11/10/2021	Poultry	-	Hatchery	A	SAMN46989061	SRR32473923
**21ER3820**	13/10/2021	Poultry	-	Broiler Producer	G	SAMN46989062	SRR32473922
**21ER3829**	11/10/2021	Poultry	-	Broiler Producer	G	SAMN46989063	SRR32473921
**21ER3891**	18/10/2021	Poultry	-	Broiler Producer	G	SAMN46989064	SRR32473919
**21ER3905**	12/10/2021	Poultry	-	Hatchery	A	SAMN46989065	SRR32473918
**21ER3912**	28/10/2021	Poultry	-	Egg Producer	E	SAMN46989066	SRR32473917
**21ER4005**	2/11/2021	Poultry	-	Broiler Producer	F	SAMN46989067	SRR32473916
**21ER4084**	5/11/2021	Poultry	-	Broiler Producer	F	SAMN46989068	SRR32473915
**21ER4096**	9/11/2021	Poultry	-	Broiler Producer	F	SAMN46989069	SRR32473914
**21ER4215**	15/11/2021	Poultry	-	Broiler Producer	F	SAMN46989070	SRR32473913
**21ER4234**	18/11/2021	Human	Manawatu	-	-	SAMN46989334	SRR32474039
**21ER4314**	22/11/2021	Poultry	-	Broiler Producer	F	SAMN46989071	SRR32473912
**21ER4337**	2/12/2021	Poultry	-	Broiler Producer	F	SAMN46989072	SRR32473911
**21ER4341**	30/11/2021	Poultry	-	Broiler Producer	F	SAMN46989073	SRR32473910
**21ER4395**	6/12/2021	Poultry	-	Broiler Producer	F	SAMN46989074	SRR32473908
**21ER4485**	10/12/2021	Poultry	-	Broiler Producer	F	SAMN46989075	SRR32473907
**21ER4519**	13/12/2021	Human	Manukau	-	-	SAMN46989335	SRR32474038
**22ER0136**	22/12/2021	Human	Auckland	-	-	SAMN46989336	SRR32474037
**22ER0144**	29/12/2021	Human	Hawke's Bay	-	-	SAMN46989337	SRR32474036
**22ER0168**	4/01/2022	Human	Hawke's Bay	-	-	SAMN46989338	SRR32474035
**22ER0173**	8/01/2022	Human	Wellington	-	-	SAMN46989339	SRR32474033
**22ER0365**	18/01/2022	Human	Taranaki	-	-	SAMN46989340	SRR32474032
**22ER0439**	28/01/2022	Poultry	-	Broiler Producer	F	SAMN46989076	SRR32473906
**22ER0484**	3/02/2022	Poultry	-	Broiler Producer	F	SAMN46989077	SRR32473905
**22ER0537**	6/01/2022	Human	Hawke's Bay	-	-	SAMN46989341	SRR32474031
**22ER0557**	4/02/2022	Human	Northland	-	-	SAMN46989342	SRR32474030
**22ER0602**	9/02/2022	Human	Auckland	-	-	SAMN46989343	SRR32474029
**22ER0655**	11/02/2022	Human	Wellington	-	-	SAMN46989344	SRR32474028
**22ER0735**	26/10/2021	Poultry	-	Broiler Producer	G	SAMN46989078	SRR32473904
**22ER0927**	15/11/2021	Poultry	-	Hatchery	A	SAMN46989079	SRR32473903
**22ER0928**	16/11/2021	Poultry	-	Hatchery	A	SAMN46989080	SRR32473902
**22ER0929**	3/12/2021	Poultry	-	Hatchery	A	SAMN46989081	SRR32473901
**22ER0932**	10/12/2021	Poultry	-	Broiler Producer	G	SAMN46989082	SRR32473900
**22ER0934**	13/12/2021	Poultry	-	Hatchery	A	SAMN46989083	SRR32473899
**22ER0939**	20/12/2021	Poultry	-	Hatchery	A	SAMN46989084	SRR32473897
**22ER0941**	29/12/2021	Poultry	-	Hatchery	A	SAMN46989085	SRR32473896
**22ER0955**	30/12/2021	Poultry	-	Hatchery	A	SAMN46989086	SRR32473895
**22ER0960**	5/01/2022	Poultry	-	Hatchery	A	SAMN46989087	SRR32473894
**22ER0961**	10/01/2022	Poultry	-	Broiler Producer	G	SAMN46989088	SRR32473893
**22ER0965**	16/01/2022	Poultry	-	Hatchery	A	SAMN46989089	SRR32473892
**22ER1062**	17/02/2022	Poultry	-	Hatchery	A	SAMN46989090	SRR32473891
**22ER1177**	14/03/2022	Poultry	-	Hatchery	A	SAMN46989091	SRR32473890
**22ER1178**	7/03/2022	Poultry	-	Hatchery	A	SAMN46989092	SRR32473889
**22ER1181**	1/03/2022	Poultry	-	Hatchery	A	SAMN46989093	SRR32473888
**22ER1184**	31/01/2022	Poultry	-	Hatchery	A	SAMN46989094	SRR32473886
**22ER1256**	20/03/2022	Poultry	-	Hatchery	A	SAMN46989095	SRR32473885
**22ER1344**	7/03/2022	Poultry	-	Hatchery	A	SAMN46989096	SRR32473884
**22ER1345**	7/03/2022	Poultry	-	Hatchery	A	SAMN46989097	SRR32473883
**22ER1346**	20/03/2022	Poultry	-	Hatchery	A	SAMN46989098	SRR32473882
**22ER1347**	22/03/2022	Poultry	-	Hatchery	A	SAMN46989099	SRR32473881
**22ER1579**	21/04/2022	Human	Manukau	-	-	SAMN46989345	SRR32474027
**22ER1697**	29/10/2021	Erinaceinae	-	Hedgehog	A	SAMN46989353	SRR32474026
**22ER1699**	2/02/2022	Erinaceinae	-	Hedgehog	A	SAMN46989354	SRR32474025
**22ER1707**	5/05/2022	Human	Manukau	-	-	SAMN46989346	SRR32474024
**22ER1758**	10/05/2022	Human	Canterbury	-	-	SAMN46989347	SRR32474022
**22ER1863**	17/05/2022	Poultry	-	Broiler Producer	D	SAMN46989100	SRR32473880
**22ER2059**	3/06/2022	Human	Canterbury	-	-	SAMN46989348	SRR32474021
**22ER2289**	4/07/2022	Poultry	-	Broiler Producer	D	SAMN46989101	SRR32473879
**22ER2493**	10/05/2022	Human	Canterbury	-	-	SAMN46989349	SRR32474020
